# The “coupler” technique for endoscopic removal of a threaded inside stent that migrated above tight hilar strictures

**DOI:** 10.1055/a-2208-5648

**Published:** 2023-12-12

**Authors:** Sho Kitagawa, Narito Murakoshi, Shori Ishikawa

**Affiliations:** 1 Gastroenterology, Sapporo Kosei General Hospital, Sapporo, Japan

Recently, encouraging data of suprapapillary inside stents for biliary strictures have been reported, and threaded inside plastic stents have been increasingly utilized to manage hilar strictures 1 2 3 . Although a knotted nylon thread is attached to the distal end, endoscopic retrieval of a completely migrated inside stent is sometimes challenging. Here we report a simple and feasible technique for the endoscopic removal of a threaded inside stent that migrated above tight hilar strictures.

A 75-year-old man with refractory hilar strictures due to IgG4-related sclerosing cholangitis developed acute cholangitis. After dilatation of hilar strictures, a 7-Fr threaded inside plastic stent (Through & Pass Inside Stent; Gadelius Medical K.K., Tokyo, Japan) was additionally placed in B6. However, the stent completely migrated into B6 when trying to place another inside stent in B8 ( Fig. 1 ). The migrated stent remained stuck regardless of how hard we pulled the retrieval thread, and due to the bent portion above the biliary strictures, none of the devices could access the stent. Therefore, we developed a removal technique, named the “coupler” technique, that uses a device delivery system with a well-tapered inner sheath (EndoSheather; Piolax Medical Devices, Kanagawa, Japan). The sheath was advanced over the guidewire to pass through the strictures. Then, after withdrawal of the inner sheath and guidewire, biopsy forceps with a 1.8-mm diameter (Radial Jaw 4P; Boston Scientific, Tokyo, Japan) were inserted through the outer sheath. Finally, while grasping the retrieval thread near the stent with the forceps, we successfully dragged the stent to the appropriate location by pulling the forceps and outer sheath ( Fig. 2 , Video 1 ).

**Fig. 1:**
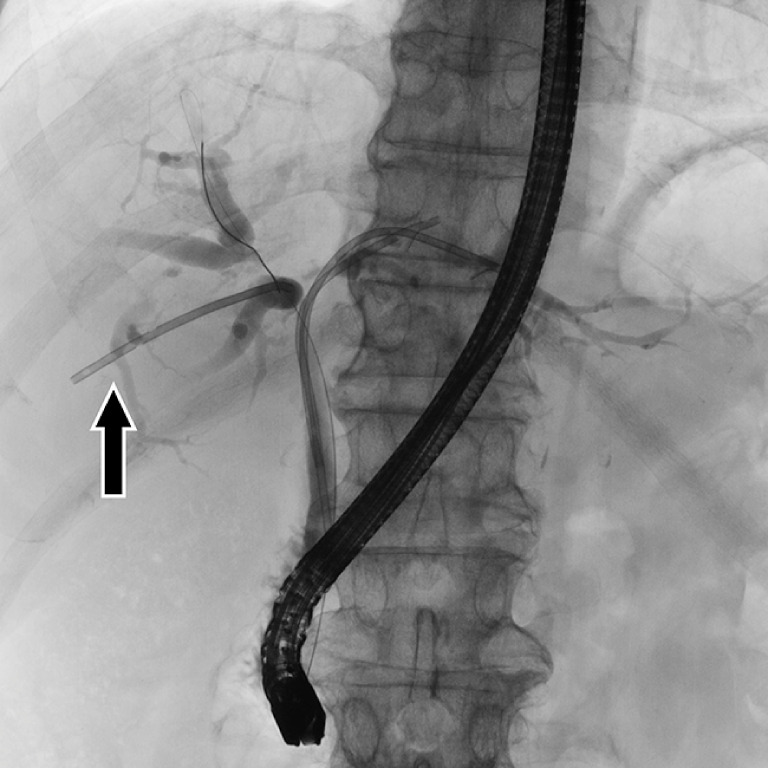
Fluoroscopic image of a 7-Fr threaded inside plastic stent that completely migrated into B6 (arrow).

**Fig. 2:**
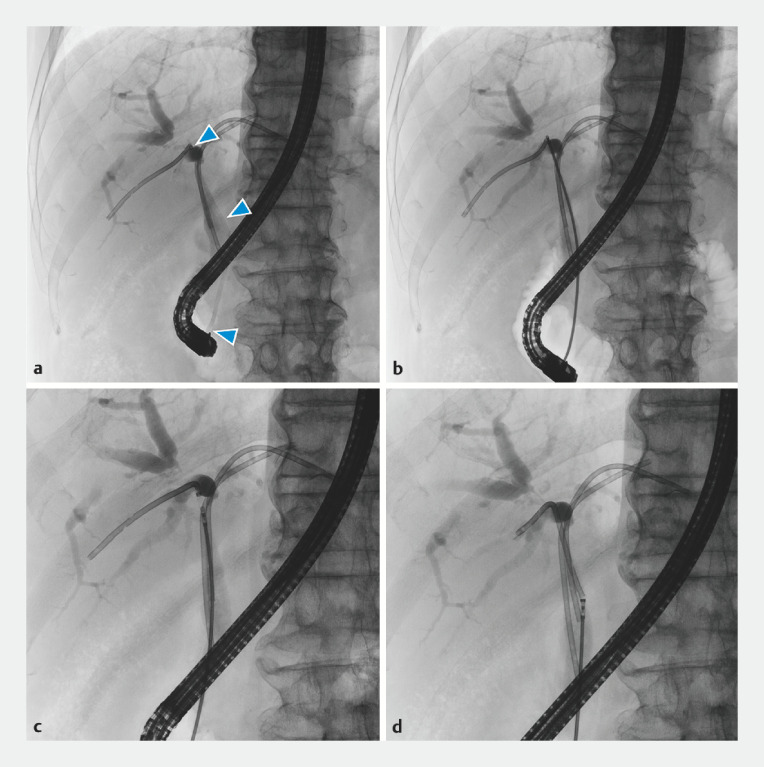
Fluoroscopic images of the procedure. **a** The outer sheath of a device delivery system (arrowheads) passing through tight hilar strictures. **b** Biopsy forceps being inserted through the outer sheath. **c** A retrieval thread near the stent being grasped with the forceps. **d** The stent being successfully dragged to the appropriate location.

The “coupler” technique for endoscopic removal of a threaded inside stent that migrated above tight hilar strictures.Video 1

In the presence of tight biliary strictures, the retrieval thread could be useless. This technique can be a helpful coping strategy for endoscopic removal of a threaded inside stent that migrated above tight hilar strictures.

Endoscopy_UCTN_Code_TTT_1AR_2AZ

## Notes

Endoscopy E-Videos https://eref.thieme.de/e-videos
*E-Videos* is an open access online section of the journal *Endoscopy* , reporting on interesting cases and new techniques in gastroenterological endoscopy. All papers include a high-quality video and are published with a Creative Commons CC-BY license. Endoscopy E-Videos qualify for HINARI discounts and waivers and eligibility is automatically checked during the submission process. We grant 100% waivers to articles whose corresponding authors are based in Group A countries and 50% waivers to those who are based in Group B countries as classified by Research4Life (see: https://www.research4life.org/access/eligibility/ ). This section has its own submission website at https://mc.manuscriptcentral.com/e-videos .
